# SSTR2 receptor states and cellular metabolism modulate ^177^Lu-DOTATATE binding affinity

**DOI:** 10.1016/j.eanmi.2026.100288

**Published:** 2026-09

**Authors:** Sara Lundsten Salomonsson, Pleun A.M. Engbers, Julie Nonnekens, Sina Bondza

**Affiliations:** a Ridgeview Instruments AB, Uppsala, Sweden; b Dept. of Immunology, Genetics and Pathology, Uppsala University, Uppsala, Sweden; c Department of Molecular Genetics, Erasmus MC Cancer Institute, Erasmus University Medical Center, Rotterdam, the Netherlands; d Department of Radiology and Nuclear Medicine, Erasmus MC Cancer Institute, Erasmus University Medical Center, Rotterdam, the Netherlands

**Keywords:** GPCR coupling, ^177^Lu-DOTATATE, SSTR2, Real-time binding kinetics, Ligand trafficking, Internalization

## Abstract

G protein-coupled receptors (GPCRs) are the target for approximately 36% of approved drugs and control many cell signaling pathways. Among these is the somatostatin receptor 2 (SSTR2), which can be targeted by the radiolabeled somatostatin analog [^177^Lu]Lu-DOTA-Tyr^3^-octreotate (^177^Lu-DOTATATE) for treatment of neuroendocrine cancers. Here, we provide a detailed kinetic characterization of the uptake and retention dynamics for ^177^Lu-DOTATATE binding to SSTR2 on live tumor cells to gain a deeper understanding on how observed binding affinities relate to receptor biology and subsequent treatment outcome.

Real-time cell binding assays have revealed two main interaction populations distinguished primarily by their retention times. High-affinity binding in the sub-nanomolar range was associated with ^177^Lu-DOTATATE interacting with G protein-coupled receptors, whereas low-affinity binding in the nanomolar range corresponded to interactions with uncoupled receptors. Cells fixed prior to ligand exposure also exhibited high-affinity binding, although to a lesser degree, implying the presence of pre-coupled SSTR2. Cellular retention decreased with ligand exposure, suggesting a shift in receptor coupling state after prolonged stimulation. Intracellular accumulation upon ligand stimulation was confirmed for both ^177^Lu-DOTATATE and SSTR2. Unlabeled DOTATATE was able to induce excretion of internalized ^177^Lu-DOTATATE, revealing a highly dynamic interaction with constant turnover of ligands.

This study highlights the dynamic nature of agonists binding to GPCRs in a live cell environment. By employing cell-based real-time binding assays, we were able de-convolute the contribution of distinct receptor states to ^177^Lu-DOTATATE affinity and retention and study the impact of internalization and recycling dynamics. Improved understanding of ligand/drug–GPCR interactions and how these are impacted by the cellular environment and metabolism can be used to optimize existing and new therapeutic approaches.

## Abbreviations

^177^Lu-DOTATATE: [^177^Lu]Lu-DOTA-Tyr^3^-octreotate
BafA: Bafilomycin A1
CHX: Cycloheximide
CV: Coefficient of variation
DOTATATE: DOTA-Tyr^3^-octreotate
FBS: Fetal bovine serum
GPCR: G protein-coupled receptor
k_a_: Association rate
k_d_: Dissociation rate
K_D_: Affinity
PDA: Polydopamine
PTX: Pertussis toxin
SD: Standard of deviation
SSTR: Somatostatin receptor
WGA: Wheat germ agglutinin

## 1 Introduction

G protein-coupled receptors (GPCRs) are the largest transmembrane protein family and are targeted by approximately 36% of approved drugs [1]. GPCRs control many signaling pathways with high precision. Despite their prominence as drug targets, not all details of the complex nature of GPCR signal transduction are fully understood [2]. Therefore, additional research on GPCRs is needed to deepen understanding of disease mechanisms and support drug development.

GPCR signaling follows a set of common principles: agonist binding induces a conformational change in the receptor allowing for GPCR coupling to intracellular heterotrimeric G proteins, which in turn can increase the affinity of the GCPR-ligand interaction [3]. GPCR signaling can be further regulated through internalization of the ligand–receptor complex, often via clathrin-coated pits, leading to depletion of receptors from the cell surface and thereby desensitization to continuous activation [4]. In contrast, antagonist ligands can bind to the receptor without activating it; i.e., it does not induce G protein activation and internalization [3].

The somatostatin receptors (SSTRs) are a subfamily of 5 GPCRs (SSTR1-5) that are expressed in the endocrine system. Despite the receptors’ involvement in cell growth suppression, SSTRs are frequently overexpressed in endocrine tumors, making them suitable targets for anti-cancer therapy [5,6]. The radiolabeled agonist [^177^Lu]Lu-DOTA-Tyr^3^-octreotate (^177^Lu-DOTATATE) is a synthetic analogue of the endogenous ligand somatostatin, which binds to SSTR2 and 5. It is approved for the targeted radionuclide therapy of gastroenteropancreatic neuroendocrine cancer [7], and is expanding into additional indications [8]. SSTR2 follows canonical GPCR signaling but exhibits ligand-dependent differences in receptor trafficking. SSTR2 is rapidly recycled to the cell membrane after stimulation with somatostatin or SSTR2-specific agonist BIM23120 [9,10], but not with octreotide [9], an agonist structurally similar to octreotate. While octreotide has been studied more extensively, the mechanisms underlying SSTR2 activation and trafficking by octreotate remain less defined, despite its wide applicability in clinical care. With several novel SSTR-targeted radiopharmaceuticals entering clinical evaluation [11], including both agonists and antagonists, understanding how synthetic ligands interact with SSTRs has become increasingly important.

The interaction of ^177^Lu-DOTATATE, or similar molecules, with SSTR2 in a cellular environment has been studied extensively with traditional end-point assays [[12], [13], [14], [15], [16], [17], [18]]. This enabled the quantification of internalized molecules but lacked the time resolution needed for calculating kinetic binding rate constants. In addition, there is evidence that kinetic studies are required to fully understand the complexity of ligand–GPCR binding and activation [19]. Kinetic characterization typically relies on real-time-based measurements, though these techniques pose their own set of challenges. Conventional real-time approaches such as quartz crystal microbalance and surface plasmon resonance typically require purified ligands [20], which is challenging when studying complex transmembrane proteins such as GPCRs that rely on membrane components for correct structure and function [21]. Therefore, time-resolved binding studies using live cells offer a more physiologically relevant alternative [[22], [23], [24], [25], [26], [27], [28]].

In this study, we performed a comprehensive characterization of ^177^Lu-DOTATATE binding and cellular trafficking, focusing on the interaction of the ligand with SSTR2 in living cancer cells. The results provide valuable insights into the dynamics of GPCR biology and can aid future development of SSTR2-based therapies.

## 2 Material and methods

### 2.1 Cell lines

U2OS cells transfected with SSTR2A (U2OS-SSTR2) were cultured in DMEM (31966-021, Thermo Fisher, Massachusetts, USA) supplemented with 10% fetal bovine serum (FBS, 10500-064, Thermo Fisher), 100 IU penicillin and 100 μg/mL streptomycin (L0022, Biowest, France) [29]. The neuroblastoma cell line IMR-32 was purchased from American Type Culture Collection (Virgina, USA) and cultured in 1:1 MEM:Ham's F12 nutrient mix (21090-022 and 11765-054, Thermo Fisher) supplemented with 10% FBS, 2 mM l-glutamine (X0550-100, Biowest), 100 IU penicillin and 100 μg/mL streptomycin.

U2OS cells transfected with SNAP-SSTR2 plasmid (U2OS-SNAP-SSTR) using a Lipofectamine 3000 kit (Thermo Fisher) [30], were cultured in DMEM (Sigma-Aldrich, MilliporeSigma, Massachusetts, USA) supplemented with 10% FBS (Serana, Germany) and 1% penicillin–streptomycin (Sigma-Aldrich, MilliporeSigma). Selection was done for 1 week using 2 mg/mL G418 (Invivogen, California, USA), after which cells were cultured in presence of 500 μg/mL G418 every other passage. Single cell subcloning was performed to obtain homogeneous colonies. Expression of SSTR2 and uptake of ^177^Lu-DOTATATE for the two U2OS-SSTR2 cell lines were verified to be of similar levels (Fig. S1, Fig. S2).

### 2.2 Chemicals, drugs and radiopharmaceuticals

#### 2.2.1 ^177^Lu-DOTATATE

In-house labeled ^177^Lu-DOTATATE or Lutathera (Advanced Accelerator Applications, France) was used for all experiments. In-house labeling was performed as previously described [31]. Briefly, 60 MBq Lutetium-177 in 0.05 M HCl (ITM, Germany) was added to 1.5 μg DOTA-Tyr^3^-octreotate (DOTATATE4046724, Bachem, Switzerland) and diluted in 25 mM sodium ascorbate/50 mM sodium acetate pH 5 (minimum volume ratio of 1:2 between HCl and Sodium ascorbate/sodium acetate) and incubated at 80^°^C for 30 min. The ^177^Lu-DOTATATE had a specific activity of 38-40 MBq/μg and a radiometal incorporation of >95%.

### 2.3 Ligand Tracer assays

Cellular uptake of ^177^Lu-DOTATATE was measured using Ligand Tracer white, yellow or grey (Ridgeview Instruments, Sweden). Default settings (2 positions, 30 s detection time, 5 s detection delay time) were used, with cells in the target area and plastic in the background area.

For U2OS-SSTR2, 0.5–2 million cells were seeded on a tilted 10 cm Petri dish and incubated at 37^°^C with 5% CO_2_ for 1–2 days prior to running assay. For IMR-32, assays were performed on cell seeded on polydopamine (PDA)-coated or Matrigel-coated petri dishes. PDA dishes were prepared by coating a part of a non-treated polystyrene petri dishes (263991, Thermo Fisher) with 600 μL 2 mg/mL PDA in 10 mM Tris pH 8 for 3 h. The PDA solution was removed, and dishes were washed twice with MQ water and dried. 1 million IMR-32 cells in PBS were added to the PDA-coated dish and incubated at room temperature for 45 min. PBS was then removed, and culture media was added. Dishes were incubated at 37^°^C for 3 days prior to running assay. For Matrigel, 400 μL 1.1% Matrigel diluted in culture media was placed on the target and background area of a cell culture Petri dish (150350, Thermo Fisher) and incubated at 37^°^C for 30–45 min. Matrigel solution was removed and 3 × 10^5^ IMR-32 cells in 400 μL culture media was placed on the area and incubated for 1-2 h, before adding 8 mL culture media and incubated 2 days.

Standard culture media or CO_2_-independent media (18045-054, Thermo Fisher) supplemented with 10% FBS, 2 mM l-glutamine, 100 IU penicillin and 100 μg/mL streptomycin was used during the Ligand Tracer assay.

Ligand Tracer assays were performed at room temperature, inside an incubator (37^°^C, 5% CO_2_) or in a cold room (7^°^C). The instrument was pre-equilibrated in the chosen assay temperature for at least 75 min. A baseline of 10–20 min was recorded, after which ^177^Lu-DOTATATE was added. One to two association phases with different ligand concentrations were recorded, followed by a dissociation phase using fresh media without ligand. For assays with slow dissociation, an additional wash step with assay media at the start of the dissociation as well as changing the assay media after approximately 30 min of dissociation was performed.

In assays where the cell-associated activity was quantified at the end of the assay, petri dishes were washed twice with PBS, and cells were detached with Trypsin–EDTA and counted. The cell-associated radioactivity was quantified using a gamma counter (Wizard^2^ 2480, Wallac, Finland).

In displacement assays, uptake of 10 nM ^177^Lu-DOTATATE to cells was monitored for 1 h, followed by removal of ^177^Lu-DOTATATE-containing media and addition of fresh media with 0.1-1 μM unlabeled DOTATATE. This approach enabled studies of ligand exchange dynamics, as displacement of ^177^Lu-DOTATATE (binding during association phase) by unlabeled DOTATATE (binding during displacement phase) resulted in a signal decrease.

### 2.4 Real-time interaction analysis

The obtained Ligand Tracer curves were processed and analyzed using TraceDrawer (1.10.1, Ridgeview Instruments) unless otherwise stated. For visual comparison, the signals were normalized at time points specified in each graph. For assays where the absolute uptake was compared, cell-associated uptake was quantified in a gamma counter at the end of the assay.

Real-time binding curves were fitted to a 1:2 kinetic model, assuming two independent 1:1 interactions with separate kinetic rate constants (association rate k_a_, dissociation rate k_d_) and affinity (K_D_) [32]. Replicates were fitted independently using default starting guesses (*k*_*a1*_ = 10^5^ M^−1^ s^−1^, *k*_*d1*_ = 3 × 10^−4^ s^−1^, *k*_*a2*_ = 3 × 10^5^ M^−1^ s^−1^, *k*_*d2*_ = 10^−3^ s^−1^) unless otherwise stated. If the initial fitting process resulted in dissociation rates below the limit of detection for the instrument (10^−7^ s^−1^), the analysis was repeated constraining the parameter to 10^−7^ s^−1^. If, upon visual inspection, there were no visible bulk effects (i.e. no signal jumps at the start of the association or dissociation phases, common artifact in real-time measurements), the BI parameter was set to 0.1. In contrast, if there were considerable concentration-dependent bulk, a 1:2 model with bulk correction was applied. The abundance of each identified interaction was quantified by comparing the *B*_max_ values:

(1) $$Abundance\;\left(\%\right)=\frac{B_{\max,1}}{B_{\max,1}+B_{\max,2}}$$

For assays where only the dissociation phase was analyzed, data was fitted to dissociation models to extract *k*_*d*_. All data were initially fitted to a 1:1 dissociation model with default starting guesses (*k*_*d*_ = 10^−3^ s^−1^). If the data had biphasic dissociation, data were analyzed with the *fit* function in Matlab (R2024b, Mathworks, Massachusetts, USA), using the built-in 1:2 dissociation model (exp*2*):

(2) $$B=B_{start,1\;}\times e^{-k_{d1}t}+B_{start,2}\times e^{-k_{d2}t}$$

Where *B* is the signal, *B*_*start,1*_ and *B*_*start,2*_ are the signals at the start of the dissociation phase for each interaction, *t* is the time and *k*_*d1*_ and *k*_*d2*_ are the dissociation rates. Starting guesses are listed in Table 1. In addition, *k*_*d2*_ was constrained to a minimum of 10^−7^ s^−1^. The abundance of each interaction was quantified by comparing the *B*_*start*_ values using equation (1).

**Table 1:** Starting guesses for 1:2 dissociation model.

Data set	*B*_*start,1*_ (% of total)	*k*_*d1*_ (s^−1^)	*B*_*start,2*_ (% of total)	*k*_*d2*_ (s^−1^)
Time series (Fig. 4)	15	10^−3^	85	10^−7^

In addition to fitting the data using kinetic models, they were analyzed using InteractionMap [33,34]. It is a tool which probes a large number of potential 1:1 interactions and returns a heat map outlining which combinations of *k*_*a*_ and *k*_*d*_ match the experimental data. Each peak on the heat map represents an interaction and the color represents the abundance (weight) of that interaction. The abundance of each identified interaction was quantified by comparing the weights using equation (1).

### 2.5 Acid wash

To quantify surface-bound ligands, acid wash with 50 mM Glycine/100 mM NaCl solution pH 2.8 was used. For more details, see Supplemental Methods.

### 2.6 Cell modification/inhibitors

For assays with fixed cells, cell dishes were incubated with 4% buffered paraformaldehyde (9713, VWR, Pennsylvania, USA) for 10 min. To inhibit the interaction between SSTR and G_i/0_ proteins, pertussis toxin (PTX, P2980, Sigma-Aldrich, MilliporeSigma) with a final concentration of 400 ng/mL and a pre-incubation of 30-90 min was used. To inhibit internalization, sucrose was added to the assay media with a final concentration of 0.5 M and a pre-incubation of 30 min. As sucrose may affect cell attachment, the cells in all assays with sucrose were counted at the end of each assay and experiments where cell detachment was higher than 20% compared to control cells were excluded from further analysis. To inhibit protein synthesis, cycloheximide (CHX, C4859, Sigma-Aldrich, MilliporeSigma) was added to the assay media with a final concentration of 100 μg/mL and a pre-incubation of 30 min. To inhibit acidification of the endosome, Bafilomycin A1 (196000, Merck, Sigma-Aldrich, MilliporeSigma) was added to the assay with a final concentration of 0.2-1 μM and a pre-incubation of 30 min.

### 2.7 Immunofluorescent staining of SSTR2

To study intracellular distribution of SSTR2, immunofluorescent staining of U2OS-SNAP-SSTR cells was performed. U2OS-SNAP-SSTR2 cells were seeded on glass coverslips in 12-well plates (5 × 10^4^ cells/well) 24h before treatment and treated with DOTATATE (10 nM) or left untreated. After 2 h, cells were fixed in 2% paraformaldehyde supplemented with 2.5 μg/mL wheat germ agglutinin 647 (WGA; W32466, Thermo Fisher) for 25 min at 4°C. For immunofluorescent staining, cells were permeabilized in PBS +0.1% Triton-x100 for 20 min. Samples were blocked for 30 min in blocking buffer (PBS, 0.5% BSA, 20 mM glycine) and incubated with SSTR2 primary antibody (Abcam, ab134152, 1:250) in blocking buffer for 1.5 h at RT. Cells were washed with PBS +0.1% Triton-x100 for 20 min, followed by incubation with secondary goat-anti-rabbit IgG Alexa Fluor 488 (A-11006, 1:1000, Thermo Fisher) in blocking buffer for 1 h at RT in the dark. Samples were incubated for 10 min in PBS with DAPI (1:1000, Thermo Fisher) and mounted on microscopic slides using vectashield (Vector Laboratories, California, USA). Imaging was performed on a SP8 confocal microscope (Leica, Microsystem, Germany) using a 40x oil immersion lens and 405 nm (DAPI), 488 nm (SSTR2) and 647 nm (WGA) lasers. Resolution was set at 1048x1048 pixels (pixel size 0.284 × 0.284 μm) and z-stacks were taken at 1 μm.

Creation of max Z-projections and manual segmentation of cellular membrane and cytoplasm were performed using Image J (version 1.53t) [35]. Segmentation of both the outer and inner membrane boundaries was done in the WGA channel, after which the raw integrated density was measured in the SSTR2 channel (Fig. S3). Clustered cells that could not be segmented were removed from the analysis, as well as cells with a total raw integrated density signal of <25000. SSTR2 signal in the cytoplasm fraction was defined as the raw integrated density within the inner membrane, whereas the membrane fraction was calculated as the raw integrated density of the area between the outer and inner membrane boundaries. The experiment was performed as 3 independent biological replicates, where each replicate contained at least 5 fields of view with ≥39 cells per condition.

### 2.8 Statistical analysis

Measured values were presented as mean with coefficient of variation (CV) in text and standard deviation (SD) in figures. Statistical analysis was performed in Graphpad Prism (8.0.1, Graphpad Software, California, USA) or Microsoft excel (Version 2509, Microsoft, Washington, USA).

## 3 Results

### 3.1 ^177^Lu-DOTATATE binding is heterogeneous and depends on G_i/o_ activity

The association and dissociation kinetics of ^177^Lu-DOTATATE to U2OS-SSTR2 cells were characterized using LigandTracer, by measuring the cell-associated radioactivity in real-time (Fig. 1, Fig. S4). The kinetics were studied at room temperature, with two uptake or association phases of 3 and 10 nM ^177^Lu-DOTATATE followed by a release or dissociation phase without ligand present.

**Fig. 1:**
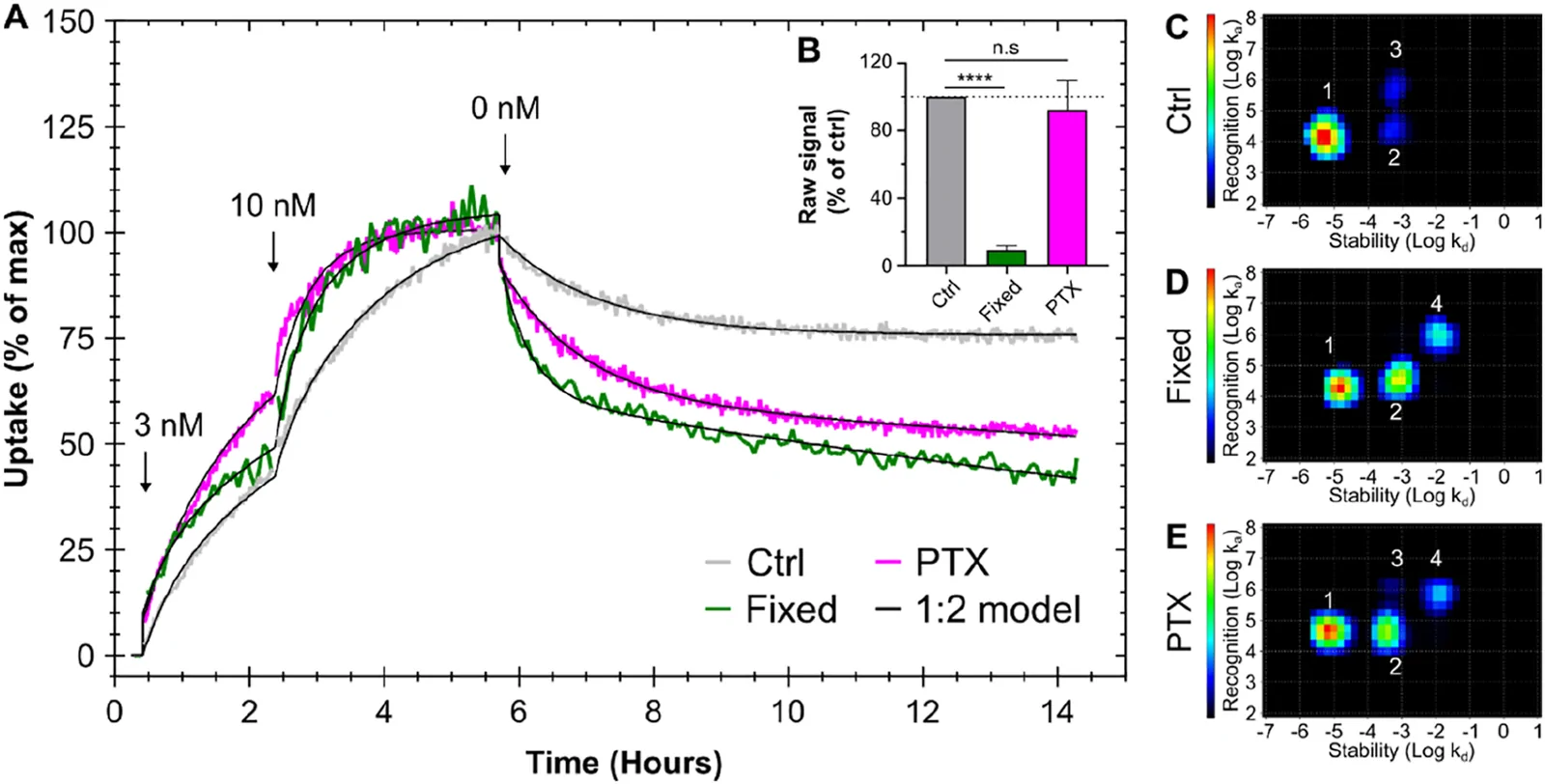
Characterization of ^177^Lu-DOTATATE binding. **A)** LigandTracer curve displaying uptake of ^177^Lu-DOTATATE to control (Ctrl), fixed or pertussis-toxin (PTX) treated U2OS-SSTR2 cells at room temperature. Curves were aligned at the start of the association phase and normalized at the end of the second association phase. Data was fitted to 1:2 binding models. **B)** The raw signal from LigandTracer traces at the end of the second association phase. Pooled data from 3 independent experiments, mean ± SD.∗∗∗∗p < 0.0001. **C-E)** InteractionMap of curves from (A), in (C) untreated, (D) fixed and (E) PTX-treated cells.

While control U2OS-SSTR2 cells had approximately 10-fold higher uptake compared to fixed cells, both binding traces displayed a bi-phasic release of ^177^Lu-DOTATATE (Fig. 1a–b, Fig. S5), i.e. an initial fast decreasing signal followed by a slower signal decrease, reflecting dissociation of tightly bound ligands. This points to the presence of (at least) two ligand-receptor populations with differing binding stability. A similar binding profile with a biphasic dissociation was also observed in IMR-32 cells that endogenously express SSTR2 (Fig. S4). To test how G protein coupling affects the binding pattern, the experiments were repeated on live cells treated with PTX, which blocks the interaction between SSTR2 and the G_i/o_ subunit (Fig. 1a, magenta curve). PTX-treated cells exhibited a faster and more pronounced bi-phasic dissociation than control cells, similar to fixed cells, but the treatment did not significantly affect the overall uptake of ^177^Lu-DOTATATE (Fig. 1b).

For all conditions, InteractionMap analysis of U2OS-SSTR2 and IMR-32 traces (Fig. 1c–e, Fig. S4b) identified two major interactions (peaks 1 and 2), separated by a difference in *k*_*d*_ but with similar *k*_*a*_ values. In control cells, InteractionMap revealed a major contribution for the stable, strong affinity interaction (peak 1), while in fixed and PTX-treated cells the abundance between strong affinity (peak 1) and weak affinity (peak 2) binding was more balanced. An additional interaction component (peak 3) was found for control cells (see Fig. S4b for IMR-32 cells) and to a lesser degree for PTX-treated cells, but absent for fixed cells. Control U2OS-SSTR2 cells had a similar abundance of peaks 2 and 3, which are very similar in their *k*_*d*_ values but differ in their *k*_*a*_. A fast-on/fast-off interaction component (peak 4) was present in fixed and PTX-treated cells, which was attributed to bulk effects arising from insufficient background subtraction (see material and methods).

As InteractionMap analysis identified two major interactions, and the data were fitted to a 1:2 model (Table 2). For U2OS-SSTR2, the two resulting association rate constants *k*_*a1*_ and *k*_*a2*_ from the 1:2 model differed 2- to 3-fold for each experimental setting, ranging from 2.19 to 7.62 M^−1^ s^−1^.

**Table 2:** Summary of kinetic analysis using a 1:2 model. Pooled data from ≥3 independent experiments, mean value and CV.

Cell line	Experimental setting	Strong affinity interaction			Weak affinity interaction			Abundance of weak affinity interaction (%)
		*k*_*a1*_ (10^4^ M^−1^ s^−1^)	*k*_*d1*_ (10^−5^ s^−1^)	*K*_*D1*_ (nM)	*k*_*a2*_ (10^4^ M^−1^ s^−1^)	*k*_*d2*_ (10^−5^ s^−1^)	*K*_*D2*_ (nM)	
U2OS-SSTR2	Control	2.93 (34%)	0.01^a^	0.004 (41%)	7.25 (45%)	21.0 (24%)	3.39 (37%)	29 (22%)
	Fixed	4.39 (30%)	1.62 (27%)	0.384 (21%)	7.62 (69%)	66.5 (38%)	15.3 (17%)	51 (23%)
	PTX	7.40 (35%)	0.47 (21%)	0.074 (13%)	2.19 (49%)	20.0 (13%)	10.85 (34%)	54 (11%)
IMR-32	Control	2.12 (21%)	0.44 (28%)	0.218 (32%)	2.61 (32%)	14.3 (6%)	6.55 (51%)	51 (22%)

a Dissociation rate was below the limit of detection and was constrained to 10^−7^ s^−1^.

In contrast to the similar association rate constants, the dissociation rate constants *k*_*d1*_ and *k*_*d2*_ differed by at least 40-fold between the two interaction components across all experimental conditions. This resulted in affinities in the sub-nanomolar range for the stable interaction (*K*_*D1*_) and the nanomolar range for the less stable interaction (*K*_*D2*_). In control cells, 29% of the total binding sites were attributed to the weak affinity interaction, whereas this proportion increased to 51% in fixed cells and 54% in PTX-treated cells. Of note, the *k*_*d1*_ in control cells was extremely slow and can effectively be interpreted as an interaction with no measurable dissociation. The same behavior, similar *k*_*a*_ but differing *k*_*d*_, was also observed in IMR-32 ctrl cells, but with a higher abundance of the weak affinity interaction (51%).

### 3.2 Internalization of ^177^Lu-DOTATATE is substantial

To understand how ligand internalization affects the interaction pattern, quantification of the internalized fraction using acid wash (Fig. 2a and b) and internalization inhibitors (Fig. 2 c and d) was performed at 37^°^C, both by manual uptake assays and using LigandTracer. The pattern of internalization followed the total uptake curve (Fig. 2a), with the internalized ligand reaching 0.46 pmol/10^5^ cells after 1 h and 0.61 pmol/10^5^ cells after 4 h.

**Fig. 2:**
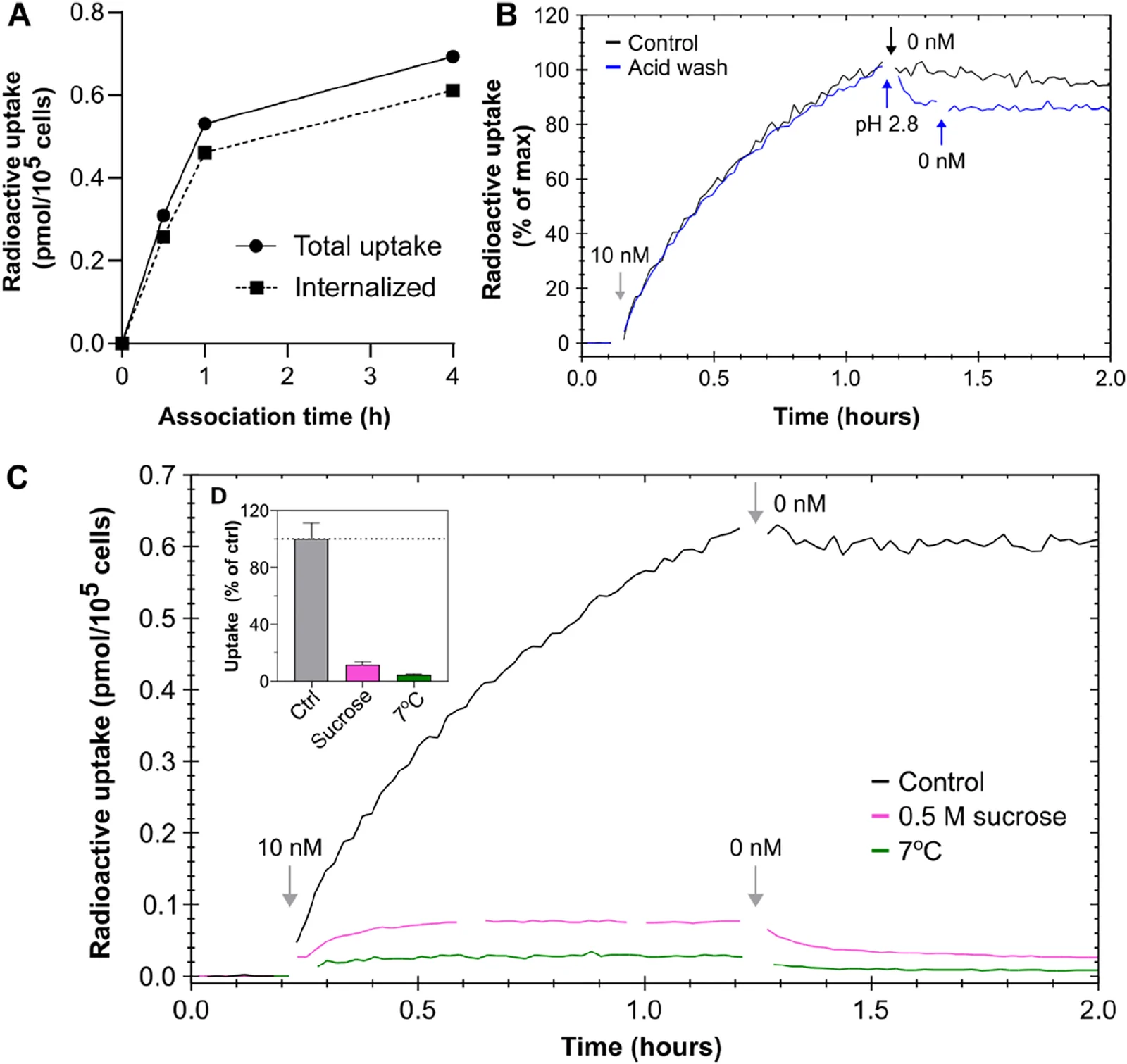
^177^Lu-DOTATATE internalization in U2OS-SSTR cells. **A)** Manual quantification of total uptake and internalized quantity of ^177^Lu-DOTATATE after incubation of 10 nM ^177^Lu-DOTATATE for 0.5-4 h. Representative experiment, mean ± SD (n = 3). **B)** Representative LigandTracer curves displaying uptake of 10 nM ^177^Lu-DOTATATE in control setting, or assay where cells were incubated with Glycine/NaCl pH 2.8. Curves were aligned at the start of the association phase and normalized at the end of the association phase. **C)** LigandTracer curves displaying uptake of 10 nM ^177^Lu-DOTATATE in control setting, media supplemented with 0.5 M sucrose, or at 7^°^C. Curves were aligned at the start of the association phase. Signal (counts per second) was converted to uptake (pmol/10^5^ cells) by counting cells and measuring cell-associated activity at the end of the experiment. **D)** The uptake from LigandTracer traces at the end of the association phase. Pooled data from 2 independent experiments, mean ± SD.

To verify these results with an orthogonal method, the binding of ^177^Lu-DOTATATE was studied in the presence of 0.5 M sucrose or at 7^°^C to inhibit internalization (Fig. 2c and d). Both setups resulted in a drastic reduction in ligand uptake after 1 h association, with a resulting total uptake of 12% (sucrose) and 4% (7^°^C) compared to control. Manual acid wash assays confirmed that the reduction in uptake was mainly due to inhibition of internalization for cells treated with sucrose, while there was a reduction in both surface-bound and internalized ligands at 7^°^C (Fig. S6). Taking all methods into account, the average internalization was 85-87% (Table 3).

**Table 3:** Summary of^177^Lu-DOTATATE internalization using acid wash or internalization inhibition with sucrose.

Method	% internalized		
	0.5h	1h	4h
Manual acid wash	76	85	87
LT acid wash	91	89	87
Sucrose^a^	88	85	-
**Average**	**85**	**86**	**87**

a It was assumed that the internalized fraction was completely abolished in sucrose-treated cells and calculations were conducted by comparing the uptake between ctrl and sucrose-treated cells.

### 3.3 Fluorescent quantification of receptor trafficking

To gain further insights into the cellular trafficking of the DOTATATE-SSTR2 complex at a single cell level, U2OS-SNAP-SSTR2 cells were incubated with 10 nM unlabeled DOTATATE for 2 h followed by fluorescent staining and imaging of SSTR2 (Fig. 3a). In untreated cells, SSTR2 was located both on the membrane and in intracellular foci. After incubation with DOTATATE, there was substantial accumulation of SSTR2 in intracellular foci, together with a decrease in the membrane-bound signal. Quantification of intracellular SSTR2 from maximum intensity projections increased from 50.2 ± 7.5% in untreated cells to 66.4 ± 7.6% in DOTATATE-treated cells (Fig. 3b). Although cells displayed varying levels of SSTR2 expression, they showed an identical internalization profile (Fig. S7).

**Fig. 3:**
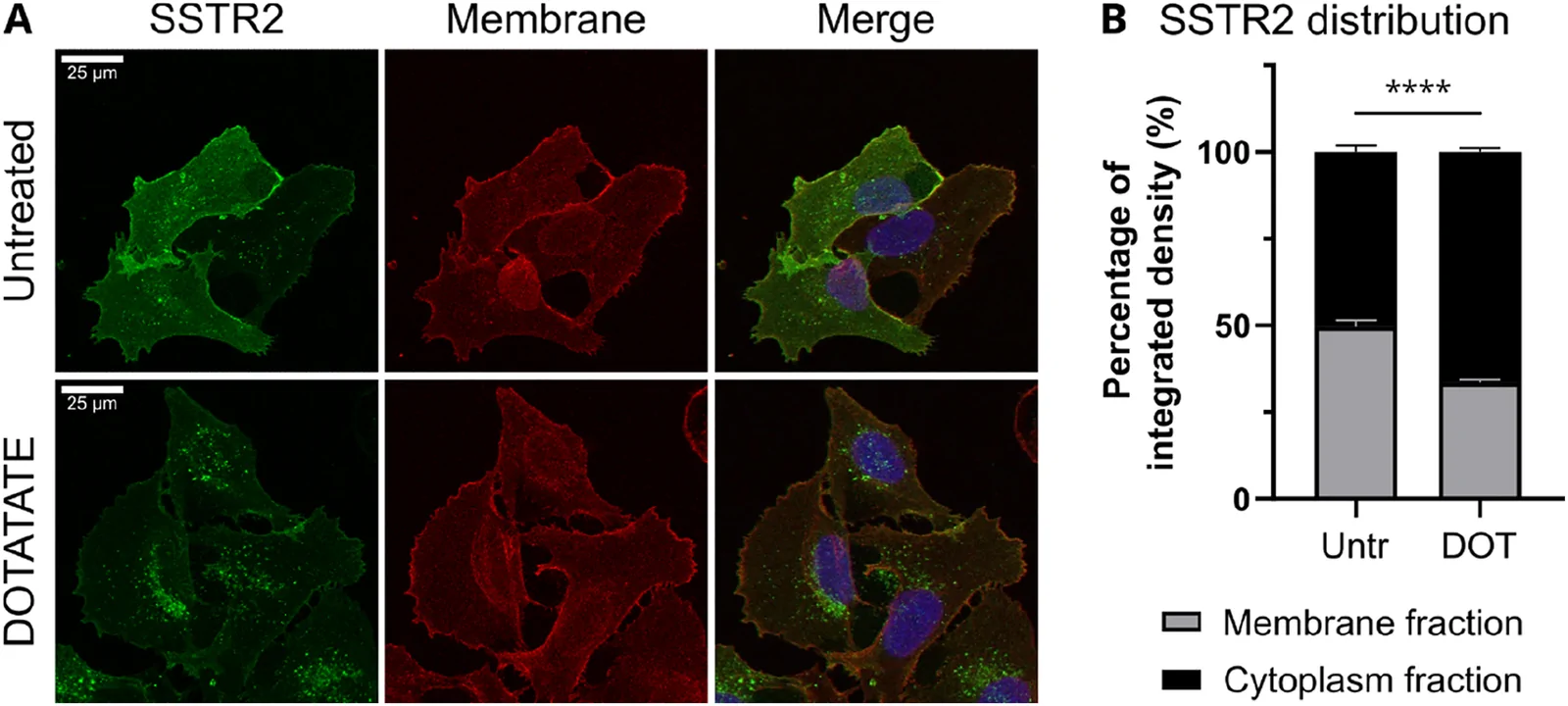
SSTR2 internalization after 2 h of DOTATATE treatment. U2OS-SNAP-SSTR2 cells were treated with DOTATATE (10 nM) for 2 h or left untreated and stained for SSTR2. **A)** Representative images of SSTR2 (green), membrane (red) and nucleus (blue) of both conditions. **B)** Quantification of raw integrated density signal of SSTR2 on membrane and internalized fractions. Pooled data from 3 independent experiments, mean ± SD. Statistical significance was determined by multiple unpaired Welch t-tests. ∗∗∗∗p ≤ 0.0001.

### 3.4 Dynamic shifts in affinity state populations and receptor synthesis further influence the uptake profile

To further characterize the two interaction populations identified in the kinetic analysis and to assess the behavior of internalized ligands, we examined the interaction between ^177^Lu-DOTATATE and SSTR2 using LigandTracer at 37^°^C while varying the association times (Fig. 4a).

**Fig. 4:**
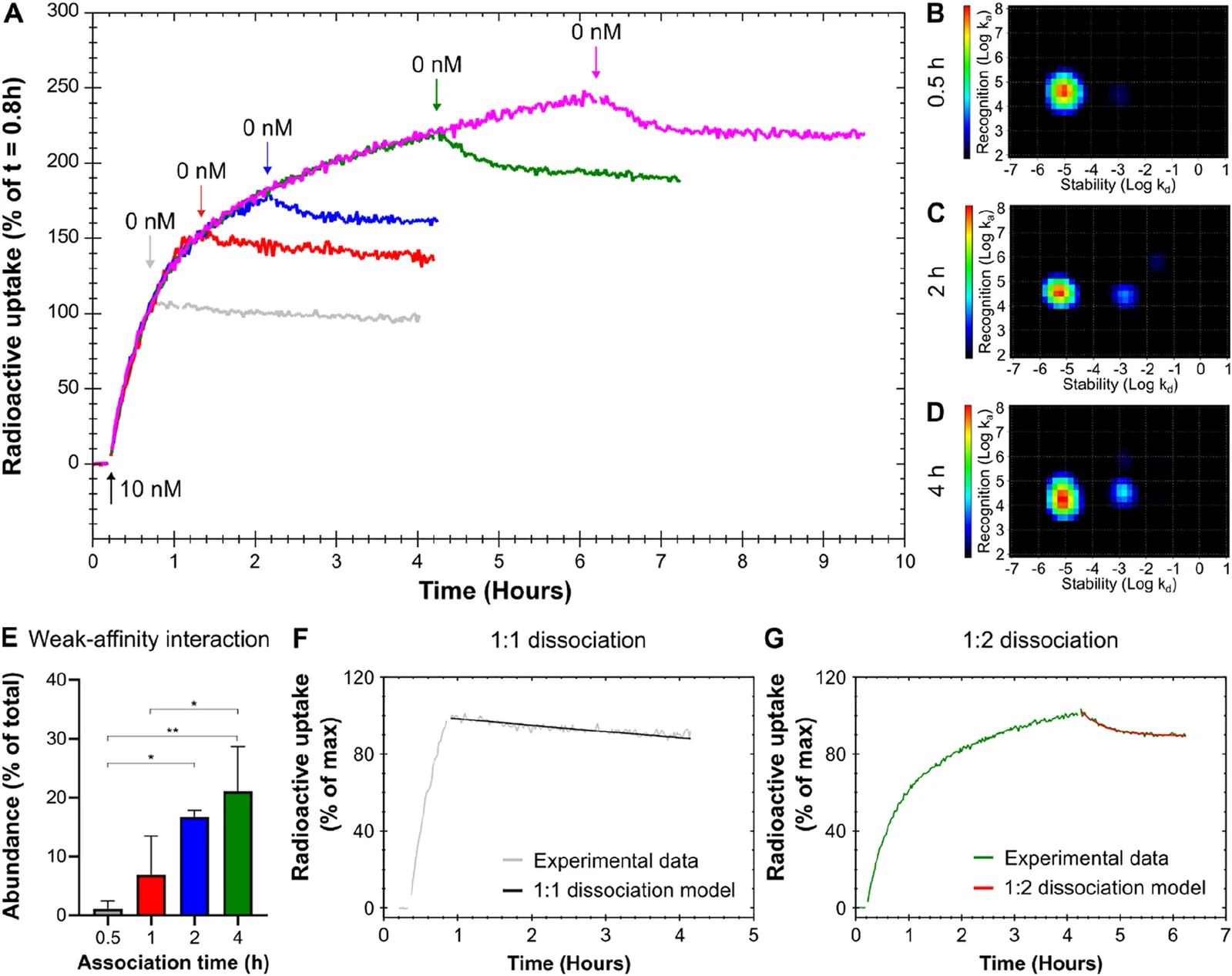
**A)** LigandTracer curves displaying uptake of 10 nM ^177^Lu-DOTATATE on U2OS-SSTR2 cells at 37^°^C, with an association time of 0.5-6 h. Black arrow: start of association phase. Colored arrows: start of dissociation phase. Curves were aligned at the start of the association phase and normalized after 30 min of association. **B-D)** InteractionMap of traces from (A), with association phase of 0.5 h (B), 2 h (C) or 4 h (D). **E)** Abundance of weak affinity interaction based on InteractionMap results. Pooled data from ≥3 independent experiments, mean ± SD. Statistical significance was determined by Tukey's *t*-test. ∗p < 0.05, ∗∗p < 0.01. Comparisons that are not displayed with significance brackets were not significant. **F-G)** Experimental data from (A) fitted to a 1:1 (F) or 1:2 (G) dissociation model.

A fast initial uptake of ^177^Lu-DOTATATE was observed, that lasted for approximately 1.5 h, followed by a seemingly linear, slower uptake that persisted for at least 4.5 h. It has been reported by others that the seemingly linear signal increase can be due to ligands binding to newly synthesized receptors [36]. To verify this, de novo protein synthesis was blocked with CHX. Indeed, the linear phase was replaced by a steady-state equilibrium in CHX-treated U2OS-SSTR2 and IMR-32 cells (Fig. S8), implying that de novo synthesis of receptors leads to additional ligand binding.

The dissociation phase displayed a slow signal decrease for shorter association times (0.5h–1h), while longer association times (2-6h) displayed a bi-phasic dissociation with an initial faster signal decrease that leveled off after approximately 1.5 h. InteractionMap analysis (Fig. 4b–d) identified two interaction populations separated by a difference in *k*_*d*_, where the abundance of the fast-off, weak affinity interaction increased with increased association time (Fig. 4e). While the weak affinity interaction component was negligible at 0.5h, it increased to 17% for 2 h association and 21% for 4h association. An increase in the fast-off, weak affinity interaction component with prolonged association times was also observed in IMR-32 cells (Fig. S9).

In line with InteractionMap results, shorter association times (0.5-1h) fitted well to a 1:1 dissociation model (Fig. 4f) with a resulting *k*_*d*_ of 1.2 × 10^−5^ s^−1^ (36%), while the 4-h curve fitted well to a 1:2 dissociation model (Fig. 4g) with resulting *k*_*d1*_ and *k*_*d2*_ values of 6.3 × 10^−4^ (21%) and 3 × 10^−7^ (116%) s^−1^, respectively. The very low *k*_*d2*_ value can in practice be interpreted as an interaction with no measurable dissociation as is the case for e.g. internalized ligands. The faster *k*_*d1*_ from the biphasic dissociation was approximately 50-fold faster than the *k*_*d*_ calculated by the 1:1 Model, which is similar to the differences in stability observed for the weak and strong affinity interactions in Table 2.

### 3.5 Ligand trafficking and recycling

To further understand the dynamics of ligand trafficking, cellular uptake of ^177^Lu-DOTATATE was measured with Ligand Tracer at 37^°^C, and during the subsequent dissociation phase unlabeled DOTATATE was added to the media. With this setup it was possible to track replacement of molecules. Binding of unlabeled DOTATATE, although not being directly detected by the instrument, reduced the signal by replacing a previously bound labeled ^177^Lu-DOTATATE.

Standard dissociation (no unlabeled ligand present) resulted in a slow signal decrease, correlating to the expected signal decrease from the release of surface-bound ligands, in comparison to the large signal decrease caused by displacement of ^177^Lu-DOTATATE by 100 nM unlabeled DOTATATE (Fig. 5 a). The number of displaced ligands was substantially greater than the surface-bound fraction, meaning that the presence of unlabeled ligands in the incubation media resulted in the expulsion of internalized ligands. Displacement data were fitted to a 1:2 model, with resulting displacement rates *k*_*dis,1*_ and *k*_*dis,2*_ of 3.4 × 10^−5^ s^−1^ (29%) and 4.5 × 10^−4^ s^−1^ (8%), respectively. The abundance of the fast-displacing ligand population (corresponding to *k*_*dis,2*_) was 75% (9%). A similar behavior was observed in IMR-32 cells (Fig. S10).

**Fig. 5:**
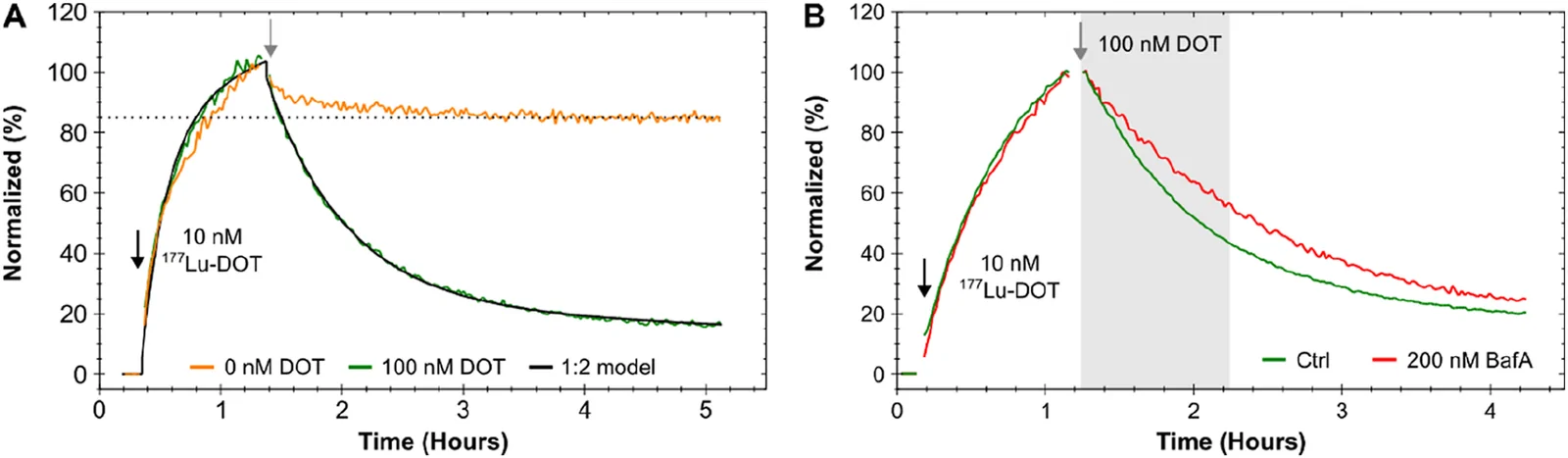
Ligand trafficking at 37^°^C. **A)** Displacement assays with 10 nM ^177^Lu-DOTATATE (^177^Lu-DOT) association for 1 h followed by displacement phase with 0 (standard dissociation setting) or 100 nM unlabeled DOTATATE (DOT) which was fitted to a 1:2 model. Traces were aligned at the start of association phase and normalized at the start of the dissociation/displacement phase. Black arrow: start of association phase. Grey arrow: start of dissociation/displacement phase. Dashed line: expected signal decrease for surface-bound ligands. Representative experiment. **B)** Displacement assays with 10 nM ^177^Lu-DOTATATE association for 1 h followed by displacement phase with 100 nM unlabeled DOTATATE in untreated cells or cells treated with 200 nM Bafilomycin A1 (BafA). Traces were aligned at the start of association phase and normalized at the start of the displacement phase. Black arrow: start of association phase. Grey arrow: start of displacement phase. Representative experiment. Data from the first hour of displacement, indicated by the grey area, was used for quantification of the initial displacement rate.

To investigate the pH dependency of the observed displacement behavior, cells were treated with BafA to block endosome acidification (Fig. 5b). Cells treated with BafA exhibited 40% slower initial displacement than untreated cells, while not affecting the relative distribution between fast and slow displacing ligand. Incubation with higher concentration of BafA did not amplify the observed effects (Fig. S11).

## 4 Discussion

The complex behavior of GPCRs and their ligands is important to understand, as GPCRs are targets for many clinically approved drugs. In this study, we performed a detailed kinetic characterization of the interaction between ^177^Lu-DOTATATE, a radiopharmaceutical approved for therapy of SSTR-expressing tumors, and the GPCR SSTR2 on cancer cells. Using real-time measurements with LigandTracer, kinetic modeling and fluorescence microscopy, we were able to quantify and separate the underlying processes contributing to the observed interaction.

The interaction between ^177^Lu-DOTATATE and SSTR2 on live cells included three main interaction populations: ligands bound to uncoupled receptors (weak affinity, nM range) and coupled receptors (strong affinity, sub-nM range) on the cell surface, as well as internalized ligands. G-protein coupling of the receptors increased the cellular retention of ^177^Lu-DOTATATE, thereby strengthening the affinity by approximately 40-fold compared to uncoupled receptors. The ability of G proteins to increase the affinity between receptor and ligand is well-established for GPCRs [3]. The fact that the strong affinity state was present in cells fixed prior to stimulation suggests that a portion of SSTRs is spontaneously coupled to G proteins in the absence of ligand, which has been described for other GPCRs [2,37]. As PTX did not completely abolish the strong affinity component, there are likely G_i/o_-independent strong-affinity interactions present on the cells, presumably due to internalized ligands (discussed further below), or receptor coupling with G protein subunits that are not affected by PTX [38].

As expected, ^177^Lu-DOTATATE internalized efficiently, with approximately 84% internalized ligands after 30 min, which is in line with previous reports [15,39,40]. Internalized ligands present an extremely slow apparent dissociation in the kinetic analysis, thus causing the observed affinity of the strong interaction in live control cells to be in the low pM range, whereas it was 0.4 nM for fixed cells that lack the internalized fraction.

In addition to the processes discussed so far, another interaction peak was detected by InteractionMap in live control cells (peak 3), characterized by a *k*_*d*_ similar to the weak affinity population but a markedly faster *k*_*a*_. Its low abundance in relation to other interaction peaks hindered further studies on its origin. Although binding of ^177^Lu-DOTATATE to other SSTR variants could account for this interaction peak, the very low uptake in untransfected U2OS cells argues against this interpretation [41]. It may be intermediate conformational states, which have been described across multiple GPCR families, or allosteric effects of G-protein coupling on the extracellular ligand-binding pocket [3].

In addition to studying the ligand behavior, we also examined the cellular distribution of SSTR2 following incubation with DOTATATE using fluorescent imaging. Consistent with previous reports [42,43], stimulation with DOTATATE increased the intracellular SSTR2 fraction and led to accumulation in intracellular foci, reflecting active internalization and trafficking. Interestingly, while the acid wash assay estimated approximately 15% surface-bound ^177^Lu-DOTATATE, imaging-based quantification suggested that 33.6% of SSTR2 were still located at the cell surface after 2h of incubation. This discrepancy likely reflects the presence of a population of unbound surface receptors, as 10 nM ^177^Lu-DOTATATE does not fully saturate SSTR2 [42]. Interpretation of imaging data was complicated by the flattened morphology of the fixed cells, which limited accurate quantification in single Z-sections. Consequently, receptor localization was evaluated using maximum-intensity projections, potentially leading to an overestimation of intracellular signal due to inclusion of top-membrane fluorescence into the cytoplasmic signal. Because this bias overestimates cytoplasmic signal, the true fraction of membrane-resident, unstimulated receptors may in fact be even higher. Despite these limitations, fluorescence microscopy enabled assessment at the single cell level with naturally varying SSTR2 expression. Importantly, this analysis revealed that differences in expression level did not influence the relative distribution of SSTR2 between membrane and cytoplasm, indicating that cells with high and low receptor abundance display comparable internalization profiles under the conditions tested.

Further characterization of the interaction at 37°C revealed that prolonged exposure to ^177^Lu-DOTATATE altered the dissociation pattern: after shorter incubation times (0.5–1h) dissociation followed a 1:1 pattern, whereas longer exposures resulted in biphasic dissociation with an increased fast-releasing (weak affinity) fraction. This time-dependent rise of weak affinity interactions suggests that more ^177^Lu-DOTATATE binds to uncoupled receptors. A plausible explanation is receptor desensitization that reduces G-protein coupling. For another GPCR, repeated activation has been shown to deplete G-protein as a desensitization mechanism [44], resulting in recycled receptors displaying weak affinity binding only.

Exposure to excess unlabeled DOTATATE revealed a rapid signal decrease far beyond the surface bound fraction, implying that internalized ^177^Lu-DOTATATE can be released from the cells. If ^177^Lu-DOTATATE remains attached to the receptor after internalization, it may tag along when the receptor is recycled back to the cell surface and subsequently be available for displacement. Blocking endosome acidification slowed the displacement of ^177^Lu-DOTATATE, indicating that it indeed reflects the recycling process. In the presence of unlabeled competitor, ^177^Lu-DOTATATE interacts with SSTR2 in a highly dynamic manner, with the constant exchange of ligands between extra- and intracellular space.

In a clinical setting, unlabeled somatostatin analogs (SSA) such as octreotide and lanreotide are used for symptom management in patients with neuroendocrine tumors [6]. Given that ^177^Lu-DOTATATE is rapidly excreted from the cells upon addition of unlabeled DOTATATE, it would be of interest to investigate whether the SSAs used in symptom management have the same effect on ^177^Lu-DOTATATE, as these are typically continued after radionuclide therapy. This is being investigated in an ongoing clinical trial (EUCT 2023-505884-35-00) [45]. A radiolabeled SSA with improved excretion properties may be able to improve therapeutic efficacy of radionuclide therapy. Excretion kinetics, together with internalization patterns and coupling states, highlight a major challenge of using agonist ligands, i.e. their complex interaction dynamics. The use of radiolabeled SSTR2 antagonists is one approach to avoid tackling this complexity [11,15,46,47], but a drawback of antagonists compared to agonists is the lack of enhanced retention by receptor coupling and internalization.

In conclusion, this study provides a detailed kinetic characterization of ^177^Lu-DOTATATE binding to SSTR2 in a live cell environment, which is impacted by receptor coupling states, internalization and recycling dynamics. The data highlights that time-resolved binding analyses on live cells enable mechanistic studies that can improve our functional understanding of ligand–receptor interactions, especially when studying complex receptors systems such as GPCRs. Improved understanding of drug–receptor interactions and how these are impacted by different factors such as addition of unlabeled competitors can be used to optimize existing and develop new therapeutic approaches.

## Written informed consent

This research did not contain human studies.

## Availability of data and material

Data is available upon reasonable request to the corresponding author.

## Ethics approval

This research did not contain human or animal studies.

## Animal welfare

This research did not involve the use of animals.

## Funding

This work was supported by ERC Starting Grant RADIOBIO 101042537 (JN).

## CRediT authorship contribution statement

**Sara Lundsten Salomonsson:** Conceptualization, Data curation, Formal analysis, Investigation, Methodology, Project administration, Visualization, Writing – original draft, Writing – review & editing. **Pleun A.M. Engbers:** Conceptualization, Formal analysis, Investigation, Methodology, Visualization, Writing – original draft, Writing – review & editing. **Julie Nonnekens:** Funding acquisition, Methodology, Project administration, Supervision, Writing – review & editing. **Sina Bondza:** Conceptualization, Formal analysis, Investigation, Methodology, Project administration, Supervision, Writing – original draft, Writing – review & editing.

## Declaration of competing interest

Sara Lundsten Salomonsson and Sina Bondza are employees of Ridgeview Instruments. For all other authors, there are no conflicts of interest.

## Data Availability

Data will be made available on request.
