# Supplementary material for: SSTR2 receptor states and cellular metabolism modulate 177Lu-DOTATATE binding affinity
Source: EANM Innov. 2026 Sep;4:None. doi: 10.1016/j.eanmi.2026.100288 (PMC13625453; doi:10.1016/j.eanmi.2026.100288)
Supplement: Supplementary file 1 [file mmc1.docx]

# Supplemental figures

**Figure S1:** Flow cytometry analysis of SSTR2 expression. Cells were stained for SSTR2 expression and SSTR2+ populations were selected in **A)** U2OS, **B)** U2OS-SSTR2 and **C)** U2OS-SNAP-SSTR2 cells. **D)** Comparison of SSTR2 expression peaks of the different cell lines.

**Figure S2.** Uptake of ^177^Lu-DOTATATE in U2OS-SSTR2 and U2OS-SNAP-SSTR2 cells. Cells were incubated with 19 nM ^177^Lu-DOTATATE for 2 h. Membrane fraction and internalized fraction were collected and normalized as the percentage added activity (AA) per 100.000 cells. Error bars represent the standard deviation (SD). Statistical significance was determined by multiple unpaired Welch t-tests. NS = not significant.

**Figure S3**. Example of manual segmentation cellular membrane and cytoplasm. Segmentation was done in WGA channel (left) and measurements were performed in SSTR2 channel (right). A: outer membrane boundary, B: inner membrane boundary, C: Region of interest (ROI) membrane fraction, D: ROI cytoplasm fraction.

**Figure S4**: Characterization of ^177^Lu-DOTATATE binding. A) LigandTracer curve displaying uptake of ^177^Lu-DOTATATE to untreated IMR-32 cells at room temperature. Data was fitted to 1:2 binding models, output is summarized in table 1. B) InteractionMap of curve from (A).

**Figure S5**: Raw signals of ^177^Lu-DOTATATE binding to U2OS-SSTR2 cells. Signals were adjusted for differences in ^177^Lu detection sensitivity between instruments.

**Figure S6:** Manual quantification of surface-bound and internalized amount of ^177^Lu-DOTATATE in U2OS-SSTR2 cells after incubation of 10 nM ^177^Lu-DOTATATE for 0.5 h. Ctrl = Control, Sucr = 0.5 M sucrose. Pooled data from two independent experiments, mean ± SD.

**Figure S7**. Differences in SSTR2 expression in U2OS-SNAP-SSTR2 cells. A-B) Representative images of SSTR2 (green), membrane (red) and nucleus (blue). A) Examples of high (h) and low (l) SSTR2 expression. B) Examples of SSTR2 positive (p) and negative (n) cells. C-D) Comparison of SSTR2 localization in cells with low, intermediate and high SSTR2 expression. Pooled data from 3 independent experiments, mean±SD. Within each fraction (cytoplasm or membrane), differences between groups were analyzed with Tukey’s multiple comparison test.

**Figure S8**: Influence of cycloheximide (CHX) on ^177^Lu-DOTATATE binding. A) LigandTracer curve displaying uptake of ^177^Lu-DOTATATE to untreated or CHX-treated U2OS-SSTR2 cells at 37^o^C. Curves were aligned at the start of the association phase and normalized after 30 min association. Representative experiment. Bar graph: manual quantification of ^177^Lu-DOTATATE uptake in untreated or CHX-treated cells after 4 h incubation. Representative experiment, mean ± SD (n=3). B) LigandTracer curve displaying uptake of ^177^Lu-DOTATATE to untreated or CHX-treated IMR-32 cells at 37^o^C. Curves were aligned at the start of the association phase and normalized after 30 min association. Representative experiment.

**Figure S9:** A) LigandTracer curves displaying uptake of 10 nM ^177^Lu-DOTATATE on IMR-32 cells at 37^o^C, with an association time of 0.5-4 h. Grey arrow: start of association phase. Colored arrows: start of dissociation phase. Curves were aligned at the start of the association phase and normalized after 30 minutes of association. B) Abundance of weak affinity interaction based on InteractionMap. Pooled data from independent experiments, mean ± SD (n≤2). C-D) InteractionMap of traces from (A), with association phase of 0.5 h (C) or 4 h (D).

**Figure S10:** Ligand trafficking at 37^o^C on IMR-32 cells. Displacement assay with 10 nM ^177^Lu-DOTATATE association for 1 h followed by displacement phase with 0 (standard dissociation setting, black) or 100 nM (blue) unlabeled DOTATATE. Traces were aligned at the start of association phase and normalized at the start of the dissociation/displacement phase.

**Figure S11:** Displacement assays on U2OS-SSTR2 cells with 10 nM ^177^Lu-DOTATATE association for 2 h followed by displacement phase with 1 µM unlabeled DOTATATE in untreated cells (black) or cells treated with 1 µM BafA (red). Traces were aligned at the start of association phase and normalized at the start of the displacement phase.

# Supplemental methods

## Acid wash

For LigandTracer assays, assays were started as above. Immediately after the association phase, the association media was removed, and cells were incubated with acid for 10 min while recording signal change. The acid was then replaced by fresh media. The ratio of surface-bound ligand was calculated as

$$R_{surface}=\frac{{{Signal}_{end assoc}-Signal}_{end acid}}{{Signal}_{end assoc}}$$

For manual assay, 1.5×10^5^ U2OS-SSTR2 were seeded in 6-well plates and incubated for 2 days. 10 nM ^177^Lu-DOTATATE was added and cells were incubated for 0.5-4 hours. Cells were washed twice with ice-cold PBS and incubated with acid for 10 min at room temperature. The acid (surface bound fraction) was collected, and cells were lysed with 0.1M NaOH (internalized fraction) for 5 minutes at room temperature. The radioactivity in each fractions was quantified using a gamma counter and representative wells with equal amounts of cells were counted.

## SSTR2 expression comparison between U2OS cells

U2OS, U2OS-SSTR2 and U2OS-SNAP-SSTR2 cells were fixed in 70% EtOH (5x10^5^ cells/tube) and permeabilized in PBS + 1% Triton-x100 for 10 min on ice. Cells were washed in PBS + 1% BSA and incubated with SSTR2 primary antibody (Abcam, ab134152, 1:100) in PBS + 1% BSA for 1.5 h at RT. Cells were washed in PBS + 0.1% Triton-x100 and incubated with secondary antibody goat-anti-rabbit IgG Alexa Fluor 488 (ThermoFisher, A-11006, 1:1000) for 1 h at RT in the dark. Samples were washed with PBS + 0.1% Triton-x100 and resuspended in PBS. SSTR2 signal was measured on a LSRFortessa flow cytometer (BD Biosciences). Data analysis was performed using FlowJo^TM^ software (BD Biosciences). Experiments were performed as 2 independent biological replicates.

## Uptake comparison between U2OS cells

U2OS-SSTR2 and U2OS-SNAP-SSTR2 cells were seeded in triplicate in 12-well plates (1x10^5^ cells/well). The next day, cells were incubated with ^177^Lu-DOTATATE in culture medium (1 MBq/mL) for 2 h at 37C. Cells were washed in PBS and membrane fraction was collected by incubation in 0.5 mL 50 mM glycine + 100 mM NaCl (pH 2.8) for 10 min at RT. Wells were washed with 0.5 mL PBS and this was added to the previously collected membrane fraction. Then, cells were lysed with 0.5 mL 0.1 M NaOH For 5 min at RT in order to collect the internalized fraction. Wells were washed with 0.5 mL PBS and added to the previously collected internalized fraction. An additional well was used to count the number of cells. Activity was measured using a 1480 automatic γ-counter (Perkin Elmer). Membrane and internalized fraction were normalized as the percentage added activity per 1x10^5^ cells. Experiments were performed as 3 independent biological replicates, each with technical triplicates.

Statistical significance for SSTR2 internalization and ^177^Lu-DOTATATE uptake was determined by multiple unpaired Welch t-tests of internalized and membrane fraction distribution between untreated and unlabeled- or ^177^Lu-DOTATATE treated cells.
